# Factors associated with appropriate home management of uncomplicated malaria in children in Kassena-Nankana district of Ghana and implications for community case management of childhood illness: a cross-sectional study

**DOI:** 10.1186/s12889-015-1777-3

**Published:** 2015-05-02

**Authors:** Soter Ameh, Paul Welaga, Caroline W Kabiru, Wilfred Ndifon, Bassey Ikpeme, Emmanuel Nsan, Angela Oyo-Ita

**Affiliations:** Department of Community Medicine, College of Medical Sciences, University of Calabar, Calabar, Cross River State Nigeria; Medical Research Council/Wits Rural Public Health and Health Transitions Research Unit (Agincourt), School of Public Health, Faculty of Health Sciences, University of the Witwatersrand, Johannesburg, South Africa; Navrongo Health Research Centre, Navrongo, Ghana; African Population and Health Research Center, Nairobi, Kenya

**Keywords:** Predictors, Barriers, Home Management of Malaria (HMM), Community Case Management of childhood illness (CCM), Children, Antimalarial drugs, Caregivers, Kassena-Nankana, Ghana

## Abstract

**Background:**

Home management of uncomplicated malaria (HMM) is now integrated into the community case management of childhood illness (CCM), an approach that requires parasitological diagnosis before treatment. The success of CCM in resource-constrained settings without access to parasitological testing significantly depends on the caregiver’s ability to recognise malaria in children under five years (U5), assess its severity, and initiate early treatment with the use of effective antimalarial drugs in the appropriate regimen at home. Little is known about factors that influence effective presumptive treatment of malaria in U5 by caregivers in resource-constrained malaria endemic areas. This study examined the factors associated with appropriate HMM in U5 by caregivers in rural Kassena-Nankana district, northern Ghana.

**Methods:**

A cross-sectional household survey was conducted among 811 caregivers recruited through multistage sampling. A caregiver was reported to have practiced appropriate HMM if an antimalarial drug was administered to a febrile child in the recommended regimen (correct dose and duration for the child’s age). Binary logistic regression was used to determine factors associated with appropriate HMM.

**Results:**

Of the 811 caregivers, 87% recognised the symptoms of uncomplicated malaria in U5, and 49% (n = 395) used antimalarial drugs for the HMM. Fifty percent (n = 197) of caregivers who administered antimalarial drugs used the appropriate regimen. In the multivariate logistic regression, caregivers with secondary (OR = 1.71, 95% CI: 1.03, 2.83) and tertiary (OR = 3.58, 95% CI: 1.08, 11.87) education had increased odds of practicing appropriate HMM compared with those with no formal education. Those who sought treatment in the hospital for previous febrile illness in U5 had increased odds of practicing appropriate HMM (OR = 2.24, 95% CI: 1.12, 4.60) compared with those who visited the health centres.

**Conclusions:**

Half of caregivers who used antimalarial drugs practiced appropriate HMM. Educational status and utilisation of hospitals in previous illness were associated with appropriate HMM. Health education programmes that promote the use of the current first line antimalarial drugs in the appropriate regimen should be targeted at caregivers with no education in order to improve HMM in communities where parasitological diagnosis of malaria may not be feasible.

**Electronic supplementary material:**

The online version of this article (doi:10.1186/s12889-015-1777-3) contains supplementary material, which is available to authorized users.

## Background

An estimated 207 million malaria cases and 627 000 malaria-related deaths occurred globally in 2012. Most of the deaths were among children under five years (U5), with 1300 young lives lost to malaria every day [1]. Sub-Saharan Africa (SSA) accounted for 85% of all deaths, with almost all of them occurring in U5 [1]. In Ghana, malaria accounts for more than 44% of outpatient visits and 22% of U5 mortality [2]. A quarter of deaths in U5 in Kassena-Nankana district of northern Ghana is attributable to malaria [3].

Malaria-related deaths in U5 occur within 48 hours of onset of illness [4]. Thus, one of the strategies of the Roll Back Malaria (RBM) programme is to reduce mortality in U5 through early diagnosis and treatment within 24 hours of the onset of symptoms [5]. However, poor physical access to health facilities is an obstacle to early diagnostic and curative services in rural areas in Low- and Middle-Income Countries (LMICs) [6,7].

There is evidence that presumptive treatment of malaria is more cost effective than prior microscopy or Rapid Diagnostic Test (RDT) in malaria endemic areas where at least 70% of fever is due to malaria [8,9]. In such settings, the World Health Organization (WHO) recommends the home management of uncomplicated malaria (HMM) strategy to reduce malaria-related morbidity and mortality in U5 [7,10]. HMM refers to the diagnosis based on the recognition of symptoms of malaria and treatment occurring outside the clinical setting, within, or near the home. The cornerstone of HMM implementation is the education of caregivers to enable them to recognise malaria, assess its severity, and initiate early treatment for uncomplicated malaria in the home using effective medicines that can be obtained from a community resource person, sales outlet, or health facility [7]. In Kassena-Nankana district, health care workers advised caregivers on how to manage uncomplicated malaria at home and to bring their children to the health facilities if they noticed any danger signs of complicated malaria [11].

Following implementation of the HMM strategy, non-prescribed antimalarial drugs became more freely available from private drug stores [12-15] without a regulatory mechanism [11,16]. In Ghana, 90% of children with fever are treated at home with drugs that are relatively more accessible from drug vendors compared to health facilities [7]. Drugs bought over the counter contributed to inappropriate treatment of febrile illness in children [17] resulting in consequent treatment failures and drug resistance [18]. Although Ghana implemented the use of Artemesinin-Based Combination Therapy (ACT) as a first line drug in 2006 [2,19], the use of non-prescribed antimalarial drugs, some of which are non-ACTs, is still high [11,20,21]. When ACTs are available, they are also dispensed based on the presumed diagnosis of malaria [20,22,23].

In an effort to more effectively regulate the use of antimalarial drugs, the HMM strategy is now integrated within the platform of the Community Case Management of childhood illness (CCM) [24]. This integration requires that trained community-based providers and volunteers, such as Community Health Workers (CHWs) and private vendors perform parasitological diagnosis before initiation of treatment with first line antimalarial drugs in the community [24]. However, in settings where parasitological diagnosis is not possible (e.g. in the home), the decision to provide antimalarial treatment must be based on the prior probability of the illness being malaria [24]. Available evidence indicates there is potential for the CCM to substantially increase access to life-saving malarial diagnostics and treatment in rural African communities [23,25,26]. The feasibility and acceptability of the CCM in urban African settings have also been described as positive [27].

However, the success of the CCM must be considered with cautious optimism. One study in Senegal found that one-third of patients who received ACTs through Lay Health Workers (LHWs) and Community Medicine Distributors (CMDs) in a rural setting had a negative Rapid Diagnostic Test (RDT), or did not receive RDT at all [23]. This finding suggests that presumptive treatment of malaria remains common practice even where parasitological diagnosis is available within the CCM strategy. A similar study in five urban sites in Ghana, Burkina Faso, Ethiopia and Malawi showed that CMDs were not universally utilised because they did not integrate well with the communities they served due to other conflicting sources of health care and lack of remuneration or incentives. Although all the five sites reported an improvement in preference for CMDs as providers of malaria treatment, the use of the current first line antimalarial drugs for self-treatment among the respondents increased in four of the five sites [27].

In order to better understand the implementation of the CCM in settings with no access to parasitological diagnosis of malaria, it is important to highlight the factors that may influence the presumptive treatment of febrile illness in U5 at home by caregivers. This paper presents the findings of a study conducted before the integration of the HMM into the CCM. More specifically, we reported knowledge of malaria, the appropriate regimen of antimalarial drugs used for the HMM in U5, barriers to uptake of HMM, and factors associated with appropriate HMM in U5 by caregivers in rural Kassena-Nankana district, northern Ghana.

## Methods

### Study setting

This study was conducted in Kassena-Nankana district, northern Ghana. The district occupies an area of 1,675 square kilometres along the Ghana-Burkina Faso border. The study area has a population of approximately 157,000 people, with females and males accounting for 52.5% and 47.5% of the population, respectively. Children under five years constitute 12.5% of the total population [28]. Agriculture is the mainstay of the district’s economy, accounting for about 69% of the employable population. In the district, there are two seasons: wet (May to October) and dry (November to April). Malaria transmission occurs throughout the year due to the all year-round warm conditions and irrigational farming which promote mosquito breeding. Malaria is the leading cause of U5 deaths in the area. Other major causes of U5 deaths include diarrhoeal diseases and acute respiratory infection, e.g. pneumonia. There is one referral district hospital, six health centres, and over 30 Community Health Centres in Kassena-Nankana district [28].

In the HMM strategy, the home is the first “hospital” for U5 in a rural community like the Kassena-Nankana district that has little or no access to diagnostic services and appropriate treatment within 24 hours of the onset of symptoms [7]. The key components of the HMM strategy were communication regarding behavioural change, training of service providers, making drugs available in the communities, and supervision and monitoring. Of these, making antimalarial drugs available in the communities, training of private drug vendors and the development of informational, educational, and communication materials were the components of the HMM strategy that were implemented in Kassena-Nankana district at the time this study was conducted. The Healthier Happier Home (He Ha Ho) campaign developed by the Ghana service used the mass and print media to target drug vendors and women’s groups in the dissemination of information on the appropriate management of malaria in U5 at home [7].

The study area was the site for a large-scale community-based intervention trial known as the Navrongo Community Health and Family Planning project. Findings from this intervention led to the Ghanaian health care policy known as the Community-based Health Planning and Services (CHPS). The CHPS concept involves relocating trained community nurses, referred to as Community Health Officers (CHOs), to rural communities to provide basic primary health care services such as routine vaccination, monitoring childhood growth and development, providing oral rehydration therapy, and health education [28]. The Navrongo Health Research Centre (NHRC) maintains a Health and Demographic Surveillance System (HDSS) which provides baseline data of all compounds, households, and individuals in the study area. All households are visited once every four months where demographic data such as births, deaths and in- and out-migration data are updated. For the purposes of conducting research, the area has been divided into five zones: East, West, North, South and Central [28]. The Navrongo HDSS database was used for sampling of prospective participants in this study.

### Study design and sampling

This was a cross-sectional study of 811 female caregivers of U5 conducted in Kassena-Nankana district between January and June 2008.

### Inclusion and exclusion criteria for recruitment of caregivers for interviews

Caregivers were interviewed in households where children manifested with fever and other symptoms of uncomplicated malaria two weeks before the survey. One caregiver per household was selected for the interview even if two or more children had fever in a household. On the other hand, caregivers were excluded from the interviews in households where children had symptoms of complicated malaria (e.g. convulsion and unconsciousness), which should be managed in health facilities as specified in the HMM strategy.

### Sample size estimation

Assuming 50% prevalence of the use of antimalarial drugs for the home management of uncomplicated malaria in U5 by caregivers in Kassena-Nankan district [29], this study had a power of 90% to identify an odds ratio of 2 as significant at 5% level of significance [30]. The minimum sample size of at least 392 caregivers was then estimated after accounting for 10% non-response. However, the sample size was doubled to 800 during data collection in order to generally understand caregivers’ health-seeking behaviour for the treatment of malaria, other than the use of antimalarial drugs. The regression analysis was then restricted to the caregivers who used antimalarial drugs for the home management of malaria in U5.

### Sampling technique

**Figure 1:**
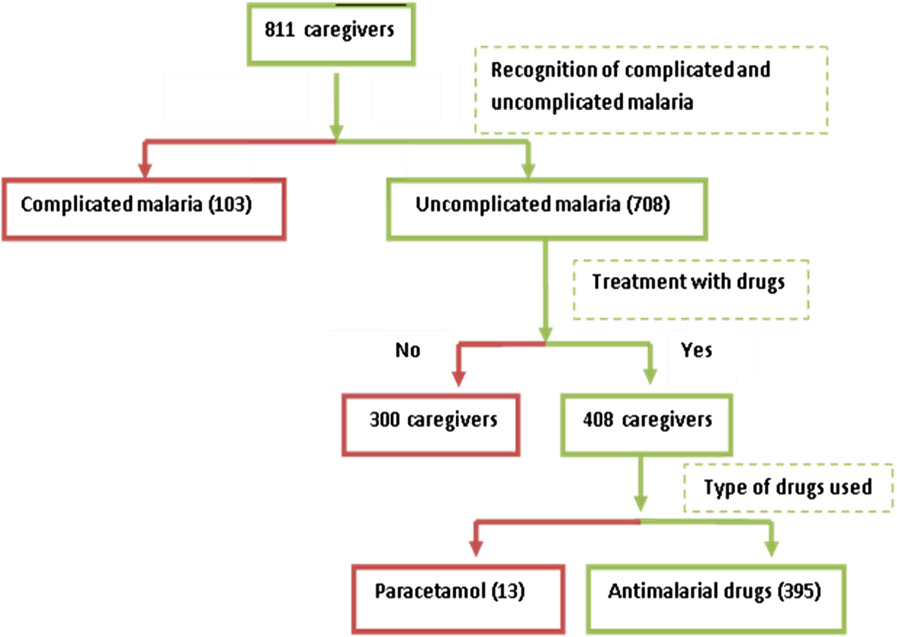
Distribution of the study participants by malaria cases reported.

Caregivers were recruited through a multistage sampling technique. In the first stage, ten communities from each of the five zones in the district were randomly selected. In the second stage, twelve compounds were randomly selected from each of the selected communities. In the third stage, all households in the sampled compounds where a child was reported to have had fever two weeks preceding the interview were selected. A caregiver in the selected households was interviewed using a semi-structured questionnaire that elicited information on socio-demographic variables; knowledge of the symptoms and treatment of uncomplicated malaria in children (Figure 1); autonomy of initiating treatment in the household; reception of advice; and sources of health care in previous febrile illness in U5.

### Training and quality control

Field workers and supervisors were recruited and trained for five days prior to data collection. Similar to another study which assessed community perceptions of malaria and malaria treatment behaviour in a rural district of Ghana [31], the field workers in our study received training on how to guide caregivers to identify antimalarial drugs that were administered to children by showing them (caregivers) pictures or packs of antimalarial drugs commonly used in Ghana. Field workers also observed left over antimalarial packs, if any, in the homes to identify antimalarial drugs that were administered by the caregivers to their children. During data collection, completed interviews were checked and verified for consistency by the supervisors at the end of each day. Incomplete questionnaires were returned to the field workers for verification. The supervisors randomly selected 5% of the completed interviews and repeated the interviews with respondents to ensure that interviews were conducted appropriately and accurately. Double entry of data was conducted by two data entry clerks and verification checks were run to reconcile differences where necessary.

### Variables

The binary outcome variable was appropriate HMM, which is defined in this paper as the administration of antimalarial drugs in the recommended age-related dose and duration using the national antimalarial treatment guideline. Knowledge of the mode of transmission of malaria was categorised as: (i) accurate - if mosquito bite was the only response, (ii) fair - if mosquito bite and any other response(s) were selected and (iii) poor - if mosquito bite was not selected. Knowledge of uncomplicated malaria was based on caregivers’ recognition of fever and any of the following symptoms in a child: vomiting, abdominal pain, weakness, diarrhoea, chills, loss of appetite, yellow-coloured urine, shivering and stomach ache. Knowledge of complicated malaria was based on caregiver’s identification of unconsciousness, convulsion, or fever in a child [32].

The wealth index of the caregivers was constructed from 24 variables on household possessions and housing characteristics, using the principal components analysis (PCA) technique [33]. Wealth index was categorised into quintiles in ascending order of lowest, middle low, middle, middle high, and highest socio-economic status.

Independent variables were age, education, occupation, religion and SES of the caregivers. Other variables assessed were lack of autonomy of initiating treatment for a febrile child, reception of advice, knowledge of malaria, and type of health care utilised for previous febrile illness in U5 among caregivers.

### Statistical analysis

Statistical analysis was done with STATA^R^ version 12. Mean and standard deviation were calculated for continuous variables and percentages for categorical variables. We declared the data set as survey data and used the “svyset” Stata syntax in the regression analysis in order to minimise underestimation of the standard error arising from multi-stage sampling. Estimates of odds ratios and standard errors were then adjusted for the sampling design. Simple and multiple binary logistic regression models were fitted to determine factors associated with appropriate home management of malaria in U5. The relationship between each of the nine independent variables and appropriate HMM was analysed by forward selection in the unadjusted regression analysis at 5% significance level. The independent variables whose confidence intervals included the null value of one in the unadjusted analysis were excluded for analysis in the adjusted model. Interactions between independent variables were tested for. The variables that were significantly (confidence intervals excluding the null value of one) associated with appropriate HMM in the unadjusted analysis were included in the final adjusted regression model at 5% significance level.

### Ethical clearance

Ethical clearance for this study was obtained from the NHRC Institutional Review Board and the Committee for Research on Human Subjects (Medical) of the University of the Witwatersrand, Johannesburg, South Africa. Community consent was obtained from the community gatekeepers (village heads) after community entry, mobilization and sensitization were done. Individual written informed consent was then obtained from all study participants following a briefing by fieldworkers about the aim of the study and rights of study participants.

## Results

### Socio-demographic characteristics of the 811 respondents

The mean age of the caregivers was 30 (SD 7.3) years. About 98% (796) of the caregivers were the biological mothers of the children. Approximately 55% (442) of the women had no formal education and most of the women were farmers (50.4%, n = 409), married (92%, n = 745) and Christians (67%, n = 547) - (Table 1).

**Table 1:** **Socio-demographic characteristics of the caregivers (N = 811)**

**Variable**	**n (%) (95% CI)**
Age group (years)	
15-24	208 (25.7) (22.7-28.8)
25-34	383 (47.2) (43.7-50.7)
≥35	220 (27.1) (24.1-30.3)
Mean age (SD) (95% CI of mean)	29.9 (7.3) (29.4-30.4)
Educational level	
No formal education	442 (54.5) (51.0-58.0)
Primary	207 (25.5) (22.6-28.7)
Secondary School	110 (13.6) (11.3-16.1)
Tertiary	52 (6.4) (4.8-8.3)
Religion	
Traditional	202 (24.9) (22.0-28.0)
Christianity	547 (67.4) (64.1-70.7)
Moslem	50 (6.2) (4.6-8.1)
Others	12 (1.5) (0.8-2.6)
Occupation	
None	144 (17.8) (15.2-20.6)
Farmer	409 (50.4) (47.0-53.9)
Trader/Civil servant	258 (31.8) (28.6-35.1)
Relationship to child	
Biological mother	796 (98.1) (97.0-99.0)
Care taker	8 (1.0) (0.4-1.9)
Grand mother	7 (0.9) (0.3-1.8)
Marital status	
Married	745 (91.9) (89.8-93.7)
Other*	66 (8.1) (6.4-10.2)
Socio-economic status	
Lowest	166 (20.5) (17.7-23.4)
Middle low	164 (20.2) (17.5-23.2)
Middle	159 (19.6) (16.9-22.5)
Middle high	176 (21.7) (18.9-24.7)
Highest	147 (18.0) (15.5-21.0)

*Other – single, divorced, separated and widowed.

### Knowledge of uncomplicated malaria and actions taken during onset of fever in U5

About 87% (708/811) of the respondents recognised the symptoms of uncomplicated malaria in U5 (Figure 1). Out of this number, 27% (189) had accurate knowledge of malaria being transmitted through mosquito bite and 53% (378) ranked fever as the cardinal symptom of uncomplicated malaria (Additional file 1). Actions taken by these caregivers to treat uncomplicated malaria were: use of herbs 69% (489), use of drugs 58% (408), sponging 24% (173), no action 2% (12) and praying/consulting soothsayers 0.4% (3) - Additional file 1.

### Drugs used for the home management of uncomplicated malaria in U5

**Figure 2:**
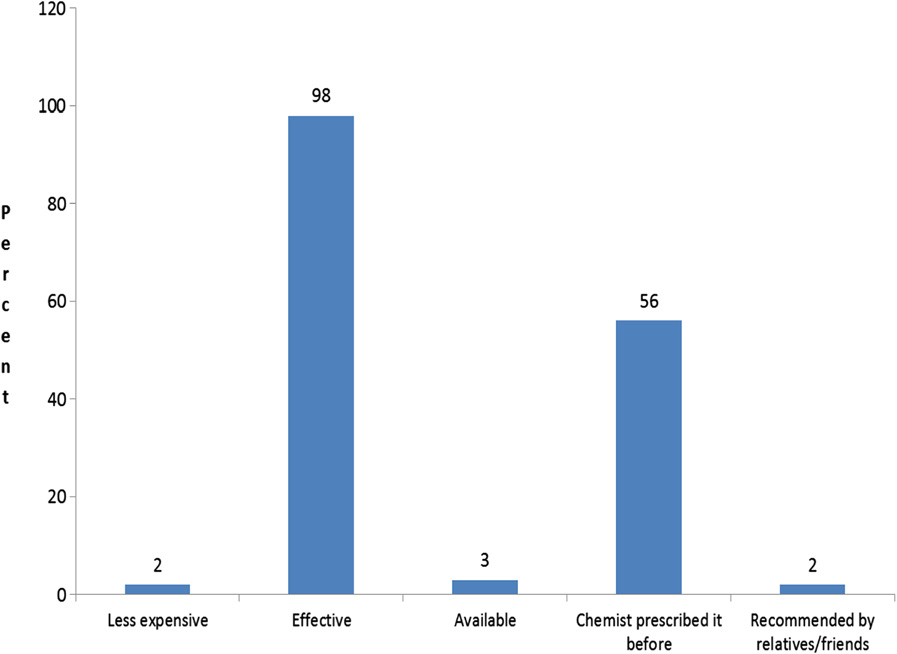
Reasons for using antimalarial drugs at home.

Table 2 shows that 395 of the 708 caregivers who recognised the symptoms of uncomplicated malaria reported using antimalarial drugs for the HMM in U5. The most common drug used was chloroquine, with 94% reported usage. Approximately 50% (197) of these caregivers administered the antimalarial drugs in the appropriate regimen. Children with febrile illness were treated at home because the caregivers perceived the drugs were effective (98%) and had previously been prescribed by a chemist or drug vendor (56%) - Figure 2.

**Table 2:** **Drugs used by caregivers for the home management of uncomplicated malaria in U5**

**Variable**	**n (%) (95% CI)**
Drugs (n = 395)	
Chloroquine	384 (94.1) (91.4-96.2)
Kinaquine®	7 (1.7) (0.7-3.5)
Fansidar®	4 (1.0) (0.3-2.5)
Use of antimalarial drugs in recommended dosage and duration	
Chloroquine (190/384)	190 (49.5) (44.4-54.6)
Kinaquine® (5/7)	5 (71.4) (29.0-96.3)
Fansidar® (2/4)	2 (50) (6.8-93.2)
All antimalarial drugs combined* (197/395)	197 (49.9) (44.8-54.9)

### Barriers to uptake of home management of malaria among the caregivers

Of the 708 caregivers who recognised the symptoms of uncomplicated malaria, 71% (501) and 92% (651) identified lack of autonomy of initiating treatment at home and reception of advice from older women as barriers to uptake of HMM, respectively (See Additional file 2). Ninety-four percent of these 651 caregivers who received advice lived in the same compound with the older women. Types of advice received were: use of herbs 84% (546), visiting the health centre/clinic 40% (263), buying drugs 24% (154) and utilising the services of the CHOs 10% (62). Ninety seven percent (629/651) of the caregivers who received advice adhered to the advice given by older women.

### Appropriate use of antimalarial drug by source of health care utilized in previous illness in U5

Table 3 shows the distribution of the 395 caregivers who used antimalarial drugs in the appropriate regimen stratified by source of health care in previous episodes of febrile illness in U5. Use of drug in the appropriate regimen was 68% (hospital), 60% (self-medication), 49% (Health centres/clinics), 48% (drug stores) and 44% (CHO’s).

**Table 3:** **Appropriate use of antimalarial drug by source of health care utilized in previous illness in U5 [n (%) (95% CI)]**

	**Health Centre/clinic**	**Drug store**	**Community Health Officers**	**Hospital**	**Self-medication**
Use of antimalarial drugs by source of health care (n = 395)	227 (57.5) (52.4-62.4)	62 (15.7) (12.2-19.7)	61 (15.4) (12.0-19.4)	40 (10.1) (7.3-13.5)	5 (1.3) (0.4-2.9)
Appropriate use of drug regimen by source of health care utilised in previous illness	110/227 (48.5) (41.8-55.2)	30/62 (48.3) (35.5-61.4)	27/61 (44.3) (31.5-57.6)	27/40 (67.5) (50.9-81.4)	3/5 (60.0) (14.7-94.7)

### Reasons for utilising sources of health care in previous febrile illness in U5

The caregivers who administered antimalarial drugs for previous febrile illness in U5 reported that effectiveness of the medication was the most common reason for utilising the hospital (88%, n = 35/40), health centres/clinics (83%, n = 188/227) and drug stores (61%, n = 38/62). On the other hand, accessibility and availability were the most common reasons for visiting the CHOs (62%, n = 38/61) and practising self-medication (40%, n = 2/5), respectively. None of the 40 caregivers who utilised the hospital for previous febrile illness indicated accessibility as the reason for the hospital visit (See Additional file 3).

### Factors associated with appropriate HMM in children with febrile illness two weeks preceding the survey

In the simple regression analysis, education, socio-economic status and type of health facility visited in previous febrile illness in U5 were significantly associated with appropriate HMM in U5 (Table 4). In the multiple regression analysis, caregivers who sought treatment in the hospital for previous febrile illness in U5 had twofold increased odds (OR = 2.24, 95% CI: 1.12, 4.60) of administering antimalarial drugs in the recommended regimen to U5 at home compared with those who utilised the community health centre/clinic. Compared with caregivers with no education, those with secondary (OR = 1.71, 95% CI: 1.03, 2.83) and tertiary (OR = 3.58, 95% CI: 1.08, 11.87) education had increased odds of administering antimalarial drugs in the recommended regimen to febrile children presumed to have uncomplicated malaria- Table 4.

**Table 4:** **Factors associated with appropriate HMM in children with febrile illness two weeks preceding the survey**

**Variable**	**Appropriate home management of malaria in U5 (N = 395)**			
	**Yes n (%)**	**No n (%)**	**Unadjusted OR (95 CI)**	**Adjusted OR (95 CI)**
	197 (49.9)	198 (50.1)		
Age group				
15-24 years	47 (23.9)	50 (25.3)	1	
25-34 years	96 (48.7)	101 (51.0)	1.01 (0.62-1.65)	
≥35 years	54 (27.4)	47 (23.7)	1.22 (0.70-2.14)	
Education				
None	100 (50.8)	130 (65.7)	1	1
Primary	60 (30.5)	39 (19.7)	0.88 (0.44-1.77)	0.59 (0.30-1.20)
Secondary	17 (8.6)	25 (12.6)	2.00 (1.24-3.23)	1.71 (1.03-2.83)
Tertiary	20 (10.1)	4 (2.0)	6.50 (2.12-19.98)	3.58 (1.08-11.87)
Occupation				
None	33 (16.7)	26 (13.2)	1	
Farmers	109 (55.0)	124 (62.9)	0.64 (0.35-1.16)	
Traders/civil servants	56 (28.3)	47 (23.9)	1.10 (0.55-2.18)	
Religion				
Traditional	49 (24.9)	57 (28.8)	1	
Christianity	137 (69.5)	124 (62.6)	1.29 (0.83-2.00)	
Moslem	11 (5.6)	17 (8.6)	0.75 (0.31-1.81)	
Marital status				
Married	183 (92.9)	180 (90.9)	1	
Other	14 (7.1)	18 (9.1)	0.77 (0.37-1.57)	
Socio-economic status (SES)				
Lowest	48 (24.4)	17 (8.6)	1	1
Middle low	48 (24.4)	45 (22.7)	0.38 (0.19-0.76)	0.40 (0.18-1.86)
Middle	36 (18.2)	42 (21.2)	0.30 (0.15-0.63)	0.34 (0.15-1.02)
Middle high	37 (18.8)	43 (21.7)	0.30 (0.15-0.62)	0.35 (0.16-1.05)
Highest	28 (14.2)	51 (25.8)	0.19 (0.09-0.40)	0.22 (0.10-1.10)
Reception of advice from older women				
No	18 (9.1)	13 (6.6)	1	
Yes	179 (90.9)	185 (93.4)	070 (0.34-1.45)	
Lack of autonomy of initiating treatment				
Yes	135 (68.5)	136 (68.7)	1	
No	62 (31.5)	62 (31.3)	0.99 (0.65-1.52)	
Source of health care utilised in the past				
Health centre/clinic	110 (55.8)	117 (59.1)	1	1
Drug store	30 (15.3)	32 (16.2)	1.02 (0.56-1.75)	1.06 (0.58-1.90)
Self-medication	3 (1.5)	2 (1.0)	0.51 (0.05-5.94)	0.51 (0.04-5.75)
CHO	27 (13.7)	34 (17.1)	0.79 (0.47-1.30)	0.78 (0.42-1.44)
Hospital	27 (13.7)	13 (6.6)	2.20 (1.09-4.49)	2.24 (1.12-4.60)

Odds ratio were adjusted in the multiple regression model for education, socio-economic status and source of health care utilised in the past.

## Discussions

In this study, we advance the state of knowledge and practice of HMM in Kassena-Nankana district and implications for the CCM strategy. Specifically, we assessed knowledge of malaria and the appropriate regimen of antimalarial drugs for the HMM in U5; barriers to uptake of HMM; and factors associated with appropriate HMM in U5 among caregivers.

The main findings in the study population showed: high knowledge of uncomplicated malaria, but fair use of antimalarial drugs in the appropriate regimen; lack of autonomy of initiation of treatment in U5 with febrile illness and reception of advice from older women were barriers to uptake of HMM; and educational status and utilisation of hospital in previous febrile illness in U5 were significantly associated with appropriate HMM in U5.

In the CCM strategy, the decision to provide antimalarial treatment in resource-constrained areas where parasitological diagnosis may not be feasible is based on the presumptive diagnosis of malaria [24]. An extensive body of literature shows that the use of antimalarial drugs by the general population is pervasive in such settings because (i) the informal drug sales outlet are often largely unregulated [16], (ii) antimalarial treatment is widely available outside the formal health settings [24] and (iii) self-treatment is common [27], possibly due to the high cost of parasitological diagnosis [24]. Therefore, the success of the CCM in resource-constrained settings will depend significantly on lay recognition of malaria, correct assessment of its severity, and early treatment of uncomplicated malaria at home using effective medicines in the appropriate regimen.

Similar to the findings of our research, previous studies conducted in Kassena-Nankana district [11,21] and the neighbouring Bolgatanga municipality [20] showed that caregivers had high knowledge of the symptoms of uncomplicated malaria in U5. Other main findings in the previous study in Kassena-Nankana district revealed that caregivers used antimalarial drugs for the HMM in U5, but not in the recommended doses [21]. Despite significant improvement in the type of drugs used by caregivers to treat malaria in U5 during the three-year period (2000–2003) that RBM tools were used to monitor progress in achieving RBM goals in Kassena-Nankana district, there was no immediate significant improvement in the dose regimen [11].

In this study, the caregivers identified lack of autonomy of decision-making in the household as a barrier to initiation of treatment at home (uptake of HMM). This notion is further supported by the fact only 29% of the caregivers who recognised the symptoms of uncomplicated malaria had the freedom of initiating treatment at home. Reception of advice from older women, most of which was on the use of herbs, was also a barrier to uptake of HMM. The predominant use of herbs by caregivers as one of the actions taken during onset of fever in U5 is a reflection of adherence to advice received from older women. Although lack of autonomy of decision-making and reception of advice were not significantly associated with appropriate HMM, interaction of these barriers could be a plausible reason for the disparity in the proportion of caregivers who recognised the symptoms of uncomplicated malaria in U5 and those who initiated treatment at home. The study in Bolgatanga municipality, which showed that the use of non-prescribed antimalarial drugs was influenced by people living around the respondents [20], further supports the role of other stakeholders in influencing the uptake of HMM by caregivers.

Health centres/clinics were the most common source of health care available to and utilised by the caregivers. This is anticipated probably due to the perceived effectiveness of antimalarial treatment received from these health facilities and the relatively high number (six CHCs and over 30 health clinics) of these facilities in the study site [28]. The foremost reason why caregivers utilised CHOs was because of their accessibility, which is the very principle for the establishment of the CHPS programme. Drug stores were perceived to be accessible, convenient, reliable, and less expensive. These attributes are also underscored in existing literature which reveal that drug stores often satisfy consumer needs for low cost and accessible health services [12-15]. The He Ha Ho communication campaign, one of Ghana’s RBM strategies to raise awareness of drug stores as a source of health care [7], may have contributed to the high use of drug stores by the caregivers.

Based on evidence from drug efficacy studies, Ghana changed its antimalarial drug policy from chloroquine to ACT as its first line drug for the treatment of malaria and HMM [19]. However, a systematic review shows that chloroquine is still being used [34,35] often in sub-optimal doses [36,37]. The use of antimalarial drugs, 99% of which was chloroquine and its brand Kinaquine®, in the appropriate regimen was 50% in this study area and higher than the 36% [38] and 16% [11] previously reported in the study site. This is expected and attributable to years of interventions targeted at improving knowledge of drug regimen in the study setting [11]. At the time this study was conducted, ACTs were the first line antimalarial drug in Ghana, but were yet to be made available in the health facilities in the study site. Even though ACTs were not administered to U5 by the caregivers in this study, information gained and lessons learned from the use of non-ACT regimen in this study are relevant and can be used to implement the CCM strategy.

Similar to a study in southwest Nigeria [39], higher educational level was associated with the use of antimalarial drugs in the appropriate regimen. In order to improve the use of antimalarial drugs in the recommended regimen at home, health education programmes that promote strict compliance to antimalarial treatment guidelines should be targeted at caregivers with little (primary) or no education in malaria endemic areas where parasitological diagnosis of malaria may be limited. In minimising barriers to uptake of HMM, health education programmes that reinforce positive health-seeking behaviour for the treatment of malaria should also be targeted at older women and other stakeholders who have considerable influence on caregivers.

Caregivers who had previously sought treatment for febrile illnesses in children in the hospital were significantly more likely to use antimalarial drugs in the appropriate regimen. This suggests that doctors working in the hospital in Kassena-Nankana district complied with protocol standards of prescription, as has been reported in Sudan [40]. The significant association between previous hospital attendance and appropriate HMM is further corroborated by the evidence that appropriate use of drug regimen by source of health care was highest (68%) for caregivers who utilised the hospital. Although accessibility of the hospital received zero ranking among the reasons for its utilisation by caregivers who used it, it is understandable due to its position as the sole referral hospital serving the district. Effectiveness of the medication prescribed by doctors in the hospital was the most common reason for its utilisation.

Our study findings should be interpreted in light of the following limitations: the lack of data on laboratory confirmation of malaria in index episode of febrile illness in U5, and self-report of fever in U5 and the use of antimalarial drugs in the recommended regimen by caregivers. However, the main strength of this research was the use of a community-based survey to identify the barriers of uptake of HMM and determine factors associated with appropriate HMM in U5 in the study population in rural Kassena-Nankana district, northern Ghana. The potential for the CCM strategy to substantially increase access to life-saving malarial diagnostics and treatment has been demonstrated [23,25-27], but presumptive self-treatment with antimalarial drugs by caregivers has also been reported in areas that the CCM strategy is being implemented [27]. The key lesson learned from this research conducted in the era of the HMM strategy is that caregivers with lower educational levels are less likely to practice appropriate HMM compared with those with higher educational level. This lesson can then be leveraged to improve the use of the current first line antimalarial drugs in the recommended regimen within the platform of the CCM strategy by targeting caregivers with little or no education in communities where parasitological diagnosis of malaria may not be feasible.

## Conclusions

In this study, half of the caregivers who recognised the symptoms of uncomplicated malaria used antimalarial drugs in the recommended regimen, according to the Ghanaian malaria treatment guideline. Lack of autonomy of initiating treatment at home and reception of advice were barriers to the uptake of HMM. Higher educational level and utilisation of hospital in previous febrile illness in U5 were associated with appropriate use of antimalarial drugs. These findings underscore the need for health education programmes that promote the use of antimalarial drugs in the appropriate regimen for the treatment of uncomplicated malaria and reinforce positive health-seeking behaviour among caregivers. These programmes must reach the wider community because in some settings, the primary caregivers may not always have the autonomy of initiating treatment.

## Additional files

Additional file 1:
**Knowledge of uncomplicated malaria and actions taken during onset of fever in U5.**
Additional file 2:
**Barriers to uptake of home management of uncomplicated malaria.**
Additional file 3:
**Reasons for utilising sources of health care by 395 caregivers who administered antimalarial drugs.**

## Abbreviations

ACT: Artemesinin-based Combination Therapy
CCM: Community Case Management of childhood illness
CHPS: Community-based Health Planning and Services
CHOs: Community Health Officers
CHWs: Community Health Workers
CMDs: Community Medicine Distributors
HDSS: Health and Demographic Surveillance System
HMM: Home Management of uncomplicated Malaria
LHWs: Lay Health Workers
LMICs: Low- and Middle-Income Countries
NHRC: Navrongo Health Research Centre
RDT: Rapid Diagnostic Test
RBM: Roll Back Malaria
SES: Socio-economic Status
SSA: Sub-Saharan Africa
U5: Children under 5 years of age
WHO: World Health Organization
